# Impact of specific training and competition on myocardial structure and function in different age ranges of male handball players

**DOI:** 10.1371/journal.pone.0143609

**Published:** 2015-12-02

**Authors:** Brahim Agrebi, Vladimir Tkatchuk, Nawel Hlila, Emna Mouelhi, Ali Belhani

**Affiliations:** 1 Department of team sports, Institute of Sports and Physical Education, Ksar Saïd, Manouba. Tunis, Tunisia; 2 Department of biological bases of physical training and sport disciplines. National University of Pedagogy Dragomanov M.P., Kiev, Ukraine; 3 Charles Nicolle hospital, Tunis, Tunisia; University of Bologna, ITALY

## Abstract

Handball activity involves cardiac changes and demands a mixture of both eccentric and concentric remodeling within the heart. This study seeks to explore heart performance and cardiac remodeling likely to define cardiac parameters which influence specific performance in male handball players across different age ranges. Forty three players, with a regular training and competitive background in handball separated into three groups aged on average 11.78±0.41 for youth players aka “schools”, “elite juniors” 15.99±0.81 and “elite adults” 24.46±2.63 years, underwent echocardiography and ECG examinations. Incremental ergocycle and specific field (SFT) tests have also been conducted. With age and regular training and competition, myocardial remodeling in different age ranges exhibit significant differences in dilatation’s parameters between “schools” and “juniors” players, such as the end-diastolic diameter (LVEDD) and the end-systolic diameter of the left ventricle (LVESD), the root of aorta (Ao) and left atrial (LA), while significant increase is observed between “juniors” and “adults” players in the interventricular septum (IVS), the posterior wall thicknesses (PWT) and LV mass index. ECG changes are also noted but NS differences were observed in studied parameters. For incremental maximal test, players demonstrate a significant increase in duration and total work between “schools” and “juniors” and, in total work only, between “juniors” and “seniors”. The SFT shows improvement in performance which ranged between 26.17±1.83 sec to 31.23±2.34 sec respectively from “seniors” to “schools”. The cross-sectional approach used to compare groups with prior hypothesis that there would be differences in exercise performance and cardiac parameters depending on duration of prior handball practice, leads to point out the early cardiac remodeling within the heart as adaptive change. Prevalence of cardiac chamber dilation with less hypertrophy remodeling was found from “schools” to “juniors” while a prevalence of cardiac hypertrophy with less pronounced chamber dilation remodeling was noted later.

## Introduction

The game of handball is characterized by actions done in a high intensity of cyclic and acyclic structures of movements performed within a wide register of technical elements, to solve problems posed by varied and complex offensive and defensive situations, after fast perception and decision making in defined space and timing [1]. The rhythm of the game imposes on handball players an intermittent type of effort which involves both isotonic and isometric forms of exercise to achieve individual and team motor skills with and without the ball. These exercises are performed primarily anaerobically, such as in accelerations, jumping and ball throwing or aerobically, in order to satisfy requests for rapid recovery between high-intensity efforts and to preserve a high level of performance during the whole match [2–4].

The impact of different sports on cardiac structure and functioning has been investigated by several authors with highly trained elite athletes [5–7]. Several studies on echocardiographic (Echo) assessment and common electrocardiographic (ECG) findings in several sports activities were conducted in individual disciplines and team sports especially in handball [8, 9], basketball [10], soccer [11], rugby [12] and volleyball [13], leading us to conclude that the structural and functional changes are of an idiosyncratic nature according to the sport discipline and the type of exercise undertaken. However, most of the collected data in these studies conducted with adult athletes have been compared to untrained controls of other sports activities. Furthermore, few investigations were dedicated to younger athletes who were still maturing physically, in spite of the morphologic changes in the heart which started in their early career. Moreover, other factors, namely age, body mass, height and training duration showed an impact on the structure and function of the heart [14–16]. That is why we found it appropriate to compare the working capacity and cardiac adaptation across normal growth and development of male preadolescent children “schools”, adolescent “juniors” and young adult “seniors” handball players using a cross-sectional approach with a priori hypothesis that there would be exercise performance and cardiac parameter differences as a function of age and corollary duration of prior handball experience.

The aim of this study is to define induced cardiac remodeling, across different age ranges in 3 groups of male handball players, observed by collecting measures and information on the state, functioning and structure changes in their hearts and to define the influence of the most important myocardial parameters on a specific field test SFT translating best the game of handball.

## Material and Methods

### Subjects

A cross-sectional approach was used to compare carefully selected 3 groups (sport categories) of Tunisian male handball players by national trainer to determine how much of the differences can be accounted for simply by normal growth and development. Weekly hours of training and competition in different age ranges increase by age. A regular week of training consisted of 1.5 h (3 times/week) and one game for “schools”; 1.5–2 h (4 times/week) and 1–2 games for “juniors” and finally, 3–4 hours daily practice sessions and 2–4 games for “seniors”. For international “juniors” and “seniors” players, more training and games (36-45/year), depending on international events, must be mentioned. The demographic **c**haracteristics, weekly training and years’ activity of all players obtained before the beginning of the experience appear in Table 1.

**Table 1:** Demographic characteristics of handball players.

Variables	Schools	Juniors	Seniors
Age (years)	11.78±0.41	15.99±0.81	24.46±2.63
Body mass (kg)	44.3±3.39 **	65.4±8.77 ††	79±4.94
Height (cm)	154±6.15 **	177.3±6.09	181.2±3.08
BMI (kg/m^2^)	18.7±1.87 *	20.8±2.27 ††	24.1±2.35
BSA (m^2^)	1.387±0.10 ***	1.813±0.26 †	1.995±0.13
Years’ activity (years)	2–3±0.7	4.5–6±1.2	12–16±2.6
Weekly training (hours)	3–4.30	6–8	12–14

BMI, body mass index; BSA, body surface area.

Schools vs. juniors:

*p<0.05;

**p<0.01;

***p<0.001

Juniors vs. seniors:

^†^p<0.05;

^††^p<0.01.

Parents and participants were informed about all procedures before obtaining written consent from subjects aged over 16 years and from parents of those under 16. The participation in this study was in accordance with the current ethical standards and this study was approved by the Ethics Committee of Tunisian National Centre of Medicine and Science in Sports.

### Procedures

The study was done in the hospital of Charles Nicolle of Tunis as well as in the High Institute of Sports and Physical Education of Tunis (Tunisia). Thirty six male players, volunteering for noninvasive cardiac testing, were examined during the competitive season (November to February 2001/2002), respecting all conditions and a schedule of 9 AM to noon. All of the participants had medical examination of the heart, lung and vascular system. In order to meet the requirements, players underwent a training program for performance enhancement and participation satisfaction. For our experiment, 43 players took part in this study and only seven of them had to be removed after receiving cardiac testing feedback or limited performance because of a previous orthopedic injury (1 school; 4 juniors and 2 seniors) which led to keep only 12 players in each age group.

2D guided M-mode and ECG measurements were determined at rest by an experienced certified cardiologist, to limit variability in data acquisition and analysis. While some functional parameters, using electromagnetically braked cycle ergometer, were assessed during maximal incremental test to define maximal workload with ECG control and blood pressure BP during effort and recuperation.

On a separate day, a validated SFT which consisted of a number of specific activities, namely a 20-m sprint, a slalom dribbling followed up by a 3-step running, a throw in the top of the goal, defensive displacements between cones, as well as a fast break followed by a jump throw in one of two lateral sides of the other goal were carried out accordingly [1]. Participants from different groups completed three trials and the best score (time in seconds) was recorded for analysis (see Table 2).

**Table 2:** Summary table of effort tests parameters.

Variables	Schools	Juniors	Seniors
HR rest (beats/min)	84.2±9.46	77.1±19.42	62.5±9.96
HR max (beats/min)	185±13.54	182.9±19.65	184.5±12.34
SBP at rest (mm Hg)	118.9±0.63*	124±10.6	126.5±1.41
SBP during effort (mm Hg)	184.1±1.97**	225.5±1.80	224±3.58
Duration of work (min)	8.6±2.54***	18.9±3.7	20.5±3.92
Total Work (Watt)	186.68±26***	356.27±30 †	438±27
Specific Field Test, SFT (sec)	31.23±1.34*	27.65±0.71 †	26.17±0.29

SBP, systolic blood pressure; HR rest, heart rate at rest; HR max, heart rate after maximum effort.

Schools vs. juniors:

*p<0.05;

**p<0.01;

***p<0.001.

Juniors vs. seniors:

^†^p<0.05.

## Methods

### Effort test

All participants performed a graded exercise test using an electromagnetically braked cycle ergometer (type Hellige Meditronic 40–3) to define the maximal workload. The frequency of cycling was 60–80 cycles a minute starting from 25 watts with an increasing load of 25 watts every 2 minutes to exhaustion. The ECG and BP are taken every 2 minutes until the end of the maximal exercise test as well as during recuperation. All procedure was supervised using a heart monitor.

### Electrocardiography

On a device of type Simens–Elema, Mingograf 82, we recorded Standard 12-lead ECG at rest in the supine position.

### Echocardiography

Using a Toshiba SSH 140A echocardiograph, simultaneity of images, in standard two-dimensional guided M-mode at rest, was collected with subjects positioned in a left lateral decubitus position, using ESC criteria and Devereux procedure [17] in order to get an acceptable level of accuracy.

### Statistical analysis

All data are expressed as mean (SD) following the changes of myocardial parameters and physical capacity with age. Comparisons between groups were performed using a one-way ANOVA and statistical significance was set at *p*< 0.05. All parameters were expressed using Principal Components Analysis (PCA) while multiple regression analyses were used to assess the influence of selected parameters on the SFT.

## Results

A number of results across and between group difference including resting and exercise hemodynamics, general work capacity, sport-specific task performance, Echo and ECG parameters are proposed to examine the cardiac structure, function, and their relationships with specific performance in handball players.

Obtained data (Table 1) show body mass index (BMI) in 3 studied groups and values that increase with age for the body surface area (BSA). Significant differences are observed in Body mass between the three studied groups while difference in height was noted only between “schools” and “juniors” at *p<0*.*01*.

### Physical tests

The fixed Total Work in different age ranges after incremental maximal exercise test performed on the cycle ergometer followed a progressive increase from “schools” 186.68±26, 356.27±30 in “juniors” to 438±27 watts in “seniors”. However the performance in SFT shows an improvement of performance through age from 31.23±1.34 sec in “schools” to 26.17±0.29 sec in “seniors”. For the HR at rest, the trend is downward while the SBP at rest increases with age (Table 2).

### Resting ECG assessment

Obtained cardiac parameters from 12-lead ECG interpreted with regard to the age are presented in Table 3. No significant differences are observed in all studied parameters between players while some changes in all values through the age are noted.

**Table 3:** Summary table of E.C.G parameters.

Variables	Schools	Juniors	Seniors
Axis QRS (°)	76°±8.43	73°±18.28	58°±30.11
Axis T (°)	75°±6.99	60.5°±20.57	55.40°±33.04
P Wave (mV)	1.10±0.46	1.40±0.52	1.50±0.47
P Wave (sec)	0.073±0.01	0.077±0.01	0.068±0.03
PR interval (sec)	0.14±0.03	0.139±0.03	0.17±0.03
SOKOLOW index (mV)	28±5.86	30.90±4.17	27.70±4.9

NS differences between “schools vs. juniors” and “juniors vs. seniors”.

### Resting Echo assessment

The quoted Echo parameters in Table 4 show that with age and after regular training and competition, handball players present morphological and functional changes within the heart as manifested by values increasing and improving. In addition, some Echo variables indexing to BSA may provide incremental value.

**Table 4:** Summary table of Echocardiographic parameters in different age ranges.

Variables	Schools	Juniors	Seniors
**Mean (SD) of cardiac values**			
LVEDD (mm)	45.50±1.06 ***	53.60±2.55	54.80±4.18
LVESD (mm)	29.26±0.40 ***	34.30±2.09	35.10±1.11
LA (mm)	27.60±0.58 *	31.30±0.66	32±2.16
Ao (mm)	23.90±0.70 **	31.50±0.34	31.10±0.64
RV (mm)	14.40±4.55	16.80±3.19	18.65±4.74
LV mass (g)	128.67±26.99 **	189.93±65.98 †	238.73±29.57
PWT (mm)	6.40±1.07	7.10±1.25 †	8.10±1.10
IVS (mm)	7.40±1.17	7.80±1.32 †††	10.35±0.89
h/R ratio	0.38±0.12	0.37±0.05	0.36±0.06
VCF (circonf/sec)	1.44±0.25	1.35±0.26	1.31±0.28
LVET (msec)	247±32.68	269±34.66	279±38.14
**Cardiac values indexed to body surface area (BSA)**			
LVEDD/BSA (mm/m^2^)	32.80±1.02 *	29.56±3.12	27.47±2.16
LVESD/BSA (mm/m^2^)	21.09±0.26 **	18.92±2.10	17.59±1.36
LA/BSA (mm/m^2^)	19.90±0,36 **	17.26±0,31	16.04±2,24
LV mass/BSA (g/m²)	92.77±20.11	104.76±15.75 †	119.66±12.67

LVEDD, end-diastolic diameter; LVESD, end-systolic diameter of the left ventricle LV; Ao, root of aorta; LA, left atrial; RV, right ventricle; VCF, mean velocity of circumferential fiber shortening; LVET, left ventricular ejection time; IVS, dimension of the inter ventricular septum; PWT, posterior wall thicknesses; h/R, LV relative wall thicknesses.

Schools vs. juniors:

*p<0.05;

**p<0.01;

***p<0.001.

Juniors vs. seniors:

^†^p<0.05;

^†††^p<0.001.

### Principal Components Analysis approach

The PCA, which total inertia reached 56.48% with all handball players, appears in the following Table 5. This saturation released the most informative myocardial and functional parameters which can be exploited as reliable parameters.

**Table 5:** Matrix of the saturations obtained by studied parameters.

Variables	FACTOR I	FACTOR II	FACTOR III
HR rest (beats-min)	- 0.05	0.39	**-0.51**
HR max (beats-min)	0.05	0.26	**-0.66**
Duration of work (min)	**0.88**	0.22	-0.12
Total work (Watt)	**0.86**	0.22	-0.10
T axis (°)	-0.32	**0.58**	-0.45
QRS axis (mV)	-0.28	0.5	**0.55**
P wave (mV)	0.24	0	**0.59**
P wave (sec)	0.31	**0.52**	0.23
PR interval (sec)	0.44	0.16	0.07
SOKOLOW index (mV)	0.16	0.02	-0.02
LVEDD (mm)	**0.86**	0.01	0.02
LVESD (mm)	**0.71**	0.18	0.25
IVS (mm)	**0.62**	-0.51	0.16
PWT (mm)	0.51	**0.70**	0.5
h/R ratio	-0.23	-0.33	0.16
VCF (circonf/sec)	-0.35	-0.14	**-0.55**
Ao (mm)	**0.71**	0.21	0.11
LA (mm)	**0.68**	-0.27	0.07
RV (mm)	0.38	0.27	0.14
TLVE (msec)	0.41	**0.57**	0.44
LV mass index (g/m²)	0.57	**-0.63**	0.17

### Stepwise multiple regression approach

To define the impact of selected parameters by PCA on the SFT in different age ranges we used the stepwise multiple regression analyses. The obtained R^2^ for all regressions is equal to 0.99 which proves furthermore that the choice of the parameters was good.

Concerning “schools”, the SFT (Y) is influenced, by the root of aorta Ao, the LV end-diastolic diameter LVEDD, the HR max reached at effort on cycle ergometer, the PR interval, the duration and the total work and by the inter ventricular septum IVS. The linear function arises as follows:

Y= 2.03*(Ao) – 0.4885*(LVEDD) + 0.0998*(HR max) + 14.96*(PR) – 0.5539*(Duration of Work) – 0.00029*(Total Work) + 0.1603*(IVS) – 12.69.

For “juniors” the selected parameters by order of importance as being able to influence SFT are the following: the PR interval, left atrial LA, LV end-systolic diameter LVESD, HR max, IVS, LV mass index and the posterior wall thicknesses PWT.

Y= 106.4*(PR) – 0.749*(LA) – 0.2107*(LVESD) + 0.1178*(HR max) + 2.1*(IVS) –0.1586*(LV mass index) + 0.9329*(PWT) + 13.74.

For the “seniors” category, we noticed that the LVEDD and the LVESD of the LV appeared as primary factors influencing the SFT, and then the PWT followed by the total and duration of work and lastly by HR max reached during the maximal exercise test.

Y= 0.7939*(LVEDD) + 0.6427*(LVESD) – 0.6582*(PWT) – 0.0017*(Total Work) + 2.142*(Duration of Work) + 0.0687*(HR max) + 24.6.

At first sight and according to the following Table 6, we notice that the HR max reflecting the cardiac answer to the maximum effort reached on the cycle ergometer is the only selected parameter which is omnipresent in the 3 proposed regressions.

**Table 6:** Ranking of selected myocardial, HR and physical parameters by stepwise analysis.

Variables	Schools	Juniors	Seniors
Ao (mm)	1	-	-
LVESD (mm)	2	-	1
**HR max** (beats-min)	**3**	**4**	**6**
PR interval (mm)	4	1	-
Duration of work (min)	5	-	5
Total work (Watt)	6	-	4
IVS (mm)	7	5	-
LA (mm)	-	2	-
LVEDD (mm)	-	3	2
LV mass index (g/m²)	-	6	-
PWT (mm)	-	7	3

LVEDD, end-diastolic diameter; LVESD, end-systolic diameter; Ao, root of aorta; LA, left atrial; IVS, dimension of the inter ventricular septum; PWT, posterior wall thicknesses.

## Discussion

Our primary goal was to define heart performance in male handball players across different age ranges. One additional goal was to identify cardiac remodeling within the heart as adaptive change. The final objective was to suggest regression models that would predict specific sport performance in different age groups. The most important finding of the present study is that regular handball activity induces cardiac remodeling primarily through cardiac dilatation in the beginning of the activity while muscular hypertrophy begins to develop some time later. Furthermore, the obtained predictive models present varied and different specific parameters according to their importance in SFT for each age range. This hints to the fact that every age range has its own idiosyncrasies mainly obtained from fluctuating parameters in regression models.

Obtained results show that the game of handball involves myocardial changes relating to the specificity of this activity which vary with age, and depend on the amount (volume) and intensity of high dynamic and moderate static forms of exercise undertaken in training/week and competitions/year on the basis of aerobic and anaerobic metabolic systems, which requests some specific morphological, functional and electrical cardiac adaptations.

### HR, Blood pressure and physical performances

At rest, the HR showed decreased values with increasing age in handball players, while displaying similar values during effort, confirming the conclusion of several authors that HR is inversely related to age and HR max is higher in children, because of their lower’s cardiac output CO and stroke volume SV, than adults [18–21].

The lower SBP response in “schools” compared to other categories is consistent with their lower SV response. The increase of the SBP with age at rest (p<0.05) and during maximal incremental exercise (p<0.01) reached between “schools-juniors”, while SBP change at rest and after exercise between “juniors-seniors” were NS. The SD of both resting HR and SBP is an order of magnitude higher for “juniors” than for either of the other groups. This suggests marked variance in this group which is not surprising as age 15–16 can place young players in very difference phases of pubertal development.

The maximal ergocycle-test (without measuring VO2 max) was geared towards assessing only the maximal workload in different age groups. The maximal workload performed on the cycle ergometer improves on average from childhood through adolescence to adult life, probably because of cumulative hours of training and competition, in addition to bio-energetic adaptations which occur during developmental years [21–23].

A significant increase of duration and total work (p<0.001) on cycle ergometer are found between “schools” and “juniors” but only in total work (p<0.05) between “juniors” and “seniors”.

Several authors found that the amount of work performed during an intense maximal test depends on glycolytic power, glycolytic capacity and aerobic ability [21]. Thus, it is necessary to pay attention when we work with children who rely more on aerobic metabolism but they demonstrate a limited energy production through anaerobic glycolysis because of their lower lactate and H^+^ production and their higher HR, breathing frequency and pH levels compared with teens and adults [19–23]. This is confirmed by the obtained results in SFT, which is considered as a high-intensity intermediate exercise requiring energy production through the anaerobic lactacid system which is limited in preadolescent children, showing a significant improvement of performance between “schools”, “juniors” and “seniors” (p<0.05).

### ECG changes

This study showed that regular participation in handball activity with regard to the athlete's age is associated effectively with ECG changes in the heart and reflecting structural and electrical remodeling [7, 24]. ECG changes in all studied parameters through the age are noted and remained within normal limits [11, 24–26]. The axis of the QRS complex is greatly dependent on age since the transition of the heart gradually shifts rightward-to-leftward with age. The screened handball players exhibit a normal QRS and T axis for their respective age [27].

The duration of PR interval represents the time between the onset of atrial depolarization and the onset of ventricular depolarization which varies according to the HR frequency and age [26, 27]. The listed PR interval measurements show a prolonged interval with age which are in normal ranges and reflect the high resting vagal tone in athletes [11, 28, 29].

The P wave which represents the atrial depolarization is influenced by the autonomic nervous system activation. Obtained values are similar to those in literature [11, 25, 26], which must be in lead V_1_ in depth (≥0.1 mV) and (≥0.04 s) in duration [24]. Lastly the results of Sokolow index, which depends of height and/or LV mass, accused also some changes with age.

### Echo changes

Several studies confirm that early myocardial remodeling is already present in pre-pubertal athletes, who have superior cardiac volume and chamber size compared with non-athletes [30–32], in spite of their short period of training and competition.

Data in different age ranges, which are in norms with the upper limit of physiologic cardiac hypertrophy, in preadolescent athletes [30], highly trained adolescent [26, 33] and highly trained elite athletes [34], confirm that with age and after regular participation in training and competition, the process of dilatation continues during the athlete's career within the heart by the increase of ventricular chamber size, which starts early in the athlete's career [35], LA, which is an additional physiological adaptation frequently present in highly trained athletes [5, 8, 36], Ao, which assure an important CO during the effort [37, 38], and IVS, PWT and LV mass index, preserving a high level of intensity required by the competition [9, 39].

The quantitative assessment of cardiac structure and function in “juniors” players compared to “schools” shows an enlarging of chamber size, increasing of muscle mass and improvement of LV performance by the increase of the left ventricular ejection time LVET, which directly varies with SV obtained by the increase of LV cavity size translating the progressive adaptation of players to increased training and competition. Furthermore the greater reliance on aerobic metabolism in young athletes must be exploited to increase cardiac size, imposing primarily a volume load on the LV, which is fundamental to improve cardiac performance particularly the SV [40–42], because it is easier to hypertrophy a dilated heart than to dilate a hypertrophied one, then continue progressively by introducing dynamic glycolytic and static exercises generating a pressure load for better forceful heart contractions to sustain a high CO during high-intensity repeated bouts [43, 44]. The data got close results to the ones obtained in other team sports as in teenage soccer players [30, 42], basketball players [10] and other disciplines [15, 31–33].

For “seniors”, the increase of the cardiac cavity size and parietal hypertrophy realize more important values compared to other categories. These findings are in accordance with those obtained in elite handball [8, 9], basketball [10], soccer players [11, 42] and other sports [32, 35, 38].

Analysis of the results shows a significant improvement of all dilatation’s parameters with a less parietal hypertrophy from “schools” to “juniors” when comparing mean values, while significant increase of parietal thickness means associated with a more or less important chamber dimensions parameters is obtained between “juniors” and “seniors”. This conclusion is confirmed by Pavlik et al. [32], suggesting that regular physical training first induces ventricular dilatation while muscular hypertrophy begins to develop some time later.

When interpreting LV mass in childhood, the American Society of Echocardiography (ASE) recommends reporting normal measurements to be indexed or divided by body surface area (BSA) in order to reduce variance among subjects [45]. Significant indexed values of Echo parameters (Table 4) concerned only three significant decreases in dilatation values between “schools” and “juniors”: LVEDD/BSA (p<0.05); LVESD/BSA (p<0.01) and LA/BSA (p<0.01). Furthermore, one significant increase of parietal thickness: LV mass/BSA (p<0.05) was observed between “juniors” and “seniors”, thus confirming the prevalence of cardiac chamber dilation in the beginning of handball activity. However a prevalence of cardiac hypertrophy was observed in young adults.

All these changes can be accepted as a physiological adaptation for systematic intense activity and to a lesser degree in other factors like genetic factors which are a subject of controversy [6, 34, 35]. This conclusion is confirmed by the result of a longitudinal study, which provided convincing evidence of the causal role between specific sport activity and cardiac structure and function [46].

### Importance of physical, Echo and ECG parameters

The interpreted Echo, ECG and physical workload changes with regard to the athlete's age show a non-linear evolution so as every age range has its peculiarities and specificities, deriving from the variations of obtained parameters in regression models.

Children exposed to handball training and competition regimens present already in early life some particular cardiac and physical parameters which can predict the SFT performance. For “schools”, the SFT performance is influenced mainly by the Ao which assure an important CO during the effort, followed by LVEDD and the HR max reached at maximal effort.

In “juniors”, the PR interval, LA, HR max, and some parietal thickness parameters such as IVS, LV mass index and PWT are selected.

For “seniors”, selected parameters can summarize the most important of them, because if LVEDD and LVESD can assure an important CO during the SFT, the PWT is necessary to sustain a high level of cardiac performance required by this test.

According to these findings, the predictive models obtained after stepwise multiple regression present varied and different specific parameters according to their importance in each age range excepting the HR max, which is present in the 3 proposed regressions.

Thus, our findings in the present study tally with the conclusions of several authors who have reported that aging changes of cardiac structure in individuals’ development are present in prepubertal children [30, 47–50], teenagers [39, 41, 51, 52] and adults [5, 8–10, 14]. Douglas and O'Toole (1985) had already demonstrated that the aging heart does manifest structural and functional changes in response to physical activities in different age groups.

## Limitations

The first limitation of this manuscript is the lack of control groups. As it stands now, the findings can indicate that age and handball practice are associated with improved performance of the myocardium, work capacity and specific tasks. Designed as an observational study and seeing how difficult it is to carry out a longitudinal study, we followed the evolution of cardiac standards across age ranges without involving control groups since researchers [8, 11, 30, 32] have already mentioned that cardiac performances are better in athletes compared with the sedentary. Nevertheless, obtained cardiac remodeling in handball players are not only due to the practice of this activity but also to other factors such as body size, genetic factors and ethnicity which should have an independent impact on tested parameters (6, 34, 35). Because our study population was limited in size and restricted to the male gender, caution should be exercised while extrapolating our findings to other ethnic and racial athletic populations. While these limitations were mostly imposed by practical issues and funding problems, this experimental study in handball team sport was difficult to put into practice.

## Conclusion

In our experimental design, we have limited our study to handball activity and age which influence cardiac structural and electrical changes in different age groups. We suggested that cardiac structure and function adaptations could occur in pre-pubertal children and continue to change through age. This is confirmed by the aforementioned authors who confirmed that the features of athletes' hearts in different age ranges, compared to control groups, are characterized by progressive and specific adaptation to regular training and competition. Thus, this study establishes reference values in different age ranges demonstrating a prevalence of cardiac chamber dilation with less cardiac hypertrophy in the beginning of handball activity between “schools” and “juniors”, while a prevalence of cardiac hypertrophy with less pronounced chamber dilation was noted later. The obtained predictive models present varied and specific parameters according to their importance for each age range.
